# Supplementary material for: Content-rich biological network constructed by mining PubMed abstracts
Source: BMC Bioinformatics. 2004 Oct 8;5:147. doi: 10.1186/1471-2105-5-147 (PMC528731; doi:10.1186/1471-2105-5-147)
Supplement: Additional File 2 — The original results of the above study (non-essential files are deleted to keep the file size under the limit set by BMC bioinformatics). [file 1471-2105-5-147-S2.bz2 › chilibotAdditionalFile2/dip05/468ID9660793E10524/html/CCL11_CXCR3.html]

 


 **CCL11** and **CXCR3** 
  
Found 87 abstracts in PubMed, retrieved 05.  
 

 What does Google say? 
 PDF only 
| .edu only 

---

**Interactive relationship** (e.g. stimulation, inhibition, etc)

**Neutral relationship**- Here, we report that although  **CCL11** , CCL24 and CCL26 are neither agonists nor antagonists of  **CXCR3** ,  **CCL11**  binds with high affinity to  **CXCR3** .  Ref: 12884299 Eur J Immunol, 2003
- This suggests that, in vivo,  **CXCR3**  may act as a decoy receptor, sequestering locally produced  **CCL11** .  Ref: 12884299 Eur J Immunol, 2003
- Eotaxin  [ **CCL11** ] , an inducer of eosinophil migration and activation, exerts its activity by binding to CCR3, the C C chemokine receptor 3  [ **CXCR3** ] .  Ref: 12857386 J Biomol Screen, 2003

**Non-interactive relationship** (e.g. studied together, co-existance, homology, etc.)

- An eosinophil active chemokine, eotaxin  [ **CCL11** ] , and its receptor, C C chemokine receptor 3  [ **CXCR3** ] , are particularly attractive as novel targets of immunological intervention for the disease.  Ref: 12842759 Cytokine< MedlineTA>Cytokine, 2003
- To study mechanisms of progenitor cell mobilization in allergic reactions, we investigated fluctuations in the expression of the eotaxin  [ **CCL11** ]  receptor, CC chemokine receptor 3  [ **CXCR3** ]  CCR3, on CD34 cells from stable asthmatics following allergeni.e. antigen challenge.  Ref: 12871220 ImmunologyImmunology, 2003
- Consistent with these results, infected cells from WT mice preferentially migrated in response to IP 10  [ **CXCR3** ]   **CXCR3**  ligand, while those from GKO mice migrated in response to eotaxin  [ **CCL11** ]   **CCL11**  CCR3 ligand.  Ref: 12875978 Am J Pathol, 2003
- Hence, eosinophil responses mediated by chemokines acting at CCR3 may be regulated by two distinct mechanisms the antagonistic effects of  **CXCR3**  ligands and the sequestration of  **CCL11**  by  **CXCR3**  expressing cells.  Ref: 12884299 Eur J Immunol, 2003
